# Green tea as an effective antimicrobial for urinary tract infections caused by *Escherichia coli*

**DOI:** 10.3389/fmicb.2013.00162

**Published:** 2013-06-18

**Authors:** Wanda Reygaert, Ilir Jusufi

**Affiliations:** ^1^Department of Biomedical Sciences, Oakland University William Beaumont School of MedicineRochester, MI, USA; ^2^Biomedical Diagnostic and Therapeutic Sciences Program, Oakland University School of Health SciencesRochester, MI, USA

**Keywords:** green tea, antimicrobial, urinary tract infections, *Escherichia coli*, antimicrobial resistance

## Abstract

**Background:** Urinary tract infections (UTIs) are a very most common type of infection worldwide, and result in billions of dollars in medical care costs. *Escherichia coli* is the infective agent for 80–90% of all UTIs. Green tea, derived from leaves of the *Camellia sinensis* plant has been shown to have various potential health benefits (e.g., cardiovascular disease and cancer). The major beneficial components of green tea have been characterized, and are now known to be polyphenolic catechins. The main catechins in green tea are (-)-epicatechin-3-gallate, (-)-epigallocatechin (EGC), (-)-epicatechin, and (-)-epigallocatechin-3-gallate (EGCG). EGCG and EGC have been shown to have the greatest antimicrobial effects, but only EGC has been shown to be excreted in urine. Isolates of *E. coli* from UTIs collected between 2007 and 2008 were characterized for antimicrobial resistance to standard drugs. Then 80 of these isolates, representing a wide spectrum of antimicrobial susceptibility patterns, were selected for testing using an extract of green tea.

**Results:** The concentrations of green tea extract tested were 0, 2.5, 3.0, 3.5, and 4.0 mg/ml. All of the strains tested, except one, had minimum inhibitory concentrations (MICs) of ≤4.0 mg/ml (99%), with 94% of the isolates having an MIC of ≤3.5 mg/ml, 76% of the isolates having an MIC of ≤3.0 mg/ml, 40% of the isolates having an MIC of ≤2.5 mg/ml. Two control strains varied in susceptibility, one having an MIC of ≤2.5 mg/ml,and the other having an MIC of ≤3.5 mg/ml.

**Conclusion:** Since EGC has been shown to have antimicrobial effects on *E. coli*, and EGC has been shown to be excreted in the urine in a high enough concentration to potentially be effective as an antimicrobial; these MIC results suggest that ingesting green tea could have potential antimicrobial effects on UTIs caused by *E. coli*.

## INTRODUCTION

Urinary tract infections (UTIs) are the second most common type of infection found in any organ system, and the most common type of nosocomial infection (Carson and Naber, 2004). These UTIs are responsible for over eight million doctors visits per year in the U.S. (National Institute of Diabetes and Digestive and Kidney Diseases, 2005), and result in medical costs of over six billion dollars worldwide per year (Anderson et al., 2004b; Kucheria et al., 2005). Most UTIs (80–90%) are the result of infections with *Escherichia coli* (Karlowsky et al., 2002). Non-pathogenic strains of *E. coli* are an important part of the normal flora in the human intestinal tract.

The strains of *E. coli* that infect the urinary tract are categorized as uropathogenic *E. coli* (UPEC; Kaper et al., 2004). The UPEC are able to produce special surface proteins (adhesins) that allow them to attach to and invade the epithelial cells that line the urinary bladder (Anderson et al., 2004a; Schaeffer et al., 2004; Kau et al., 2005; Marrs et al., 2005). If the infection is not eradicated while it is in the bladder (uncomplicated UTI), some strains of UPEC may then travel up the ureters to the kidneys and cause even more severe infections (complicated UTIs), which can lead to renal damage and possibly renal failure (Kaper et al., 2004; Pichon et al., 2009). There are 14 serogroups of UPEC that are most commonly found in UTIs, and 75% of UTIs have been shown to be caused by serogroups 04, 06, 014, 022, 075, and 083 (Stenutz et al., 2006; Li et al., 2010). The most common serogroups involved in causing UTIs worldwide are 02, 04, 06, and 075 (George and Manges, 2010). In the U.S., 49% of UTIs in women have been found to be caused by serogroups 06, 04, and 075, in descending frequency of occurrence (Vosti, 2007).

The antimicrobial agents that have traditionally been used to treat UTIs (β-lactams, fluoroquinolones, trimethoprim–sulfamethoxazole, nitrofurantoin, etc.) are becoming less effective (Warren et al., 1999; Wagenlehner and Naber, 2005). In recent years, the number of antimicrobial resistant strains of *E. coli* isolated from UTIs has been increasing, including resistance to antimicrobial agents normally used to treat UTIs (Sahm et al., 2001; Kahlmeter, 2003; Muratani and Matsumoto, 2004; Landgren et al., 2005; Zhanel et al., 2006). Even though scientists are constantly working to develop new and improved antimicrobials, almost as soon as a new drug is released, the bacteria show resistance to it. These isolates are also showing resistance to drug combinations such as amoxicillin/clavulanic acid, piperacillin/tazobactam, and trimethoprim/sulfamethoxazole (Dias et al., 2009; Gündoğdu, 2011; Jadhav et al., 2011; Molina-López et al., 2011; Oliveira et al., 2011).

Green tea is derived from non-fermented leaves of the *Camellia sinensis* plant. Oolong and black tea are made from fermented leaves of the same plant. Green tea has been a favored drink, traditionally, in Asian countries. Because of studies that have shown the potential health benefits of green tea, it is now gaining worldwide popularity as a drink that is important in preventative medicine. Studies using green tea have shown it to have potential benefits, most notably in: cardiovascular disease, cancer, diabetes, obesity, oral health, bone health, and cognitive function (McKay and Blumberg, 2002; Cabrera et al., 2006; Chacko et al., 2010; Mak, 2012). In addition, green tea has been shown to have antimicrobial effects (Yam et al., 1997; Taylor et al., 2005; Friedman, 2007; Song and Seong, 2007).

The components in green tea that are responsible for these various effects are polyphenols (also known as catechins). The main catechins in green tea are (-)-epicatechin (EC), (-)-epicatechin-3-gallate (ECG), (-)-epigallocatechin (EGC), and (-)-epigallocatechin-3-gallate (EGCG), which have been shown to make up approximately 30–40% of the water-soluble solids in brewed green tea (Wang and Ho, 2009). Three of these catechins, ECG, EGC, and EGCG have been show to have antimicrobial effects against a wide variety of microorganisms (Yam et al., 1997; Taguri et al., 2004, 2006; Taylor et al., 2005; Friedman, 2007; Song and Seong, 2007). The two found in the highest amounts in green tea are EGC and EGCG. Both of these are excreted in bile, but EGC is also excreted in the urine, suggesting the possibility for green tea having antimicrobial activity in UTIs (Yang et al., 1998; Lee et al., 2002; Cabrera et al., 2006; Luo et al., 2006). Most of the studies on how these compounds accomplish the antibacterial activity have focused on EGCG. Because EGCG is not excreted in the urine (Yang et al., 1998), it is not the compound of interest for this study. One study has found that EGC is able to bind to the ATP binding site of the bacterial gyrase B subunit, thus inhibiting gyrase activity (Gradivšar, 2007). The aim of the current study was to investigate the susceptibility of UPEC strains, representing a wide variety of antimicrobial susceptibility patterns, to green tea.

## MATERIALS AND METHODS

### BACTERIA STRAINS

The bacterial strains used in this study were part of a research collection of *E. coli* isolated from UTI cultures during the years of 2007–2008. There were 80 isolates selected from this collection that represented a wide spectrum of antimicrobial susceptibility patterns; in addition, two control strains that were susceptible to all the clinically tested (prior to this study) antimicrobials were selected. **Table 1** shows the antimicrobial agents tested and frequency of the strains that were resistance to each drug.

**Table 1:** Frequency of the antimicrobial resistance of *E. coli* strains used in this study.

Antimicrobial	Resistant, *n* (%)
Ampicillin	63 (78)
Piperacillin	57 (71)
Ampicillin/sulbactam	53 (66)
Tetracycline	35 (44)
Ciprofloxacin	33 (41)
Trimethoprim/sulfamethoxazole	29 (36)
Cefazolin	12 (15)
Gentamicin	12 (15)
Tobramycin	10 (13)
Cefuroxime	9 (11)
Amoxicillin/clavulanic acid	7 (9)
Aztreonam	5 (6)
Ceftriaxone	5 (6)
Nitrofurantoin	5 (6)
Cefepime	4 (5)
Norfloxacin	3 (4)
Piperacillin/tazobactam	2 (3)

### GREEN TEA EXTRACT

A standardized green tea (*C. sinensis*) extract (standardized to 7.0% polyphenols) was obtained from Swanson Vitamins^®^. The plant extract was prepared in water.

### MEDIA

The media used in these experiments was Luria-Bertani (LB) broth (Teknova brand), and dehydrated Müller-Hinton agar (MHA; BD brand).

### MINIMUM INHIBITORY CONCENTRATION DETERMINATION

The minimum inhibitory concentration (MIC) of the green tea extract was determined by agar dilution method. MHA plates that contained various concentrations of green tea extract (0, 2.5, 3.0, 3.5, and 4.0 mg/ml) were prepared. Suspensions of 0.5 McFarland standard dilutions (equal to 1.5 × 10^8^ cells/ml) were prepared from bacterial cultures grown overnight in LB broth. A 10^-^^4^ dilution of these suspensions was prepared, and 100 μl of dilution per plate was inoculated using a spread plate technique (plated concentration equals 1.5 × 10^3^ cells). Inoculated plates were incubated at 37° C for 48 h, and on the basis of colony counts, the MIC was determined as the lowest concentration of green tea extract to show an inhibitory effect on growth of the bacteria (no colonies detected). Each strain was tested three times.

## RESULTS

### MIC DETERMINATION

The MICs and susceptibility results are as follows: 99% were susceptible to the green tea extract at a concentration of ≤4.0 mg/ml (one strain was not susceptible at even 4.0 mg/ml); 94% were susceptible at ≤3.5 mg/ml; 76% were susceptible at ≤3.0 mg/ml; 40%, were susceptible at ≤2.5 mg/ml; The control strains varied; one being susceptible at ≤2.5 mg/ml and the other susceptible at ≤3.5 mg/ml. **Table 2** shows the MIC results for the 79 strains that were susceptible at ≤4.0 mg/ml.

**Table 2:** Minimum inhibitory concentrations for total green tea extract (GTE) and the EGC component.

Component	Number of isolates tested (% out of 79)			
	32 (40%)	29 (37%)	14 (18%)	4 (5%)
Total GTE (mg/ml)	≤2.5	≤3.0	≤3.5	≤4.0
EGCμg/ml)	≤450	≤540	≤630	≤720

## DISCUSSION

The green tea extract was shown to have an inhibitory effect on the growth of *E. coli* strains isolated from UTIs. The MIC results can be adjusted to reflect the EGC content, using a value of 18% EGC as the content in total green tea polyphenols (Vuong et al., 2011). That makes the adjusted results as follows: 40% of strains tested were susceptible at a concentration of EGC at ≤0.45 mg/ml (450 μg/ml); 36% susceptible at ≤0.54 mg/ml (540 μg/ml); 18% susceptible at ≤0.63 mg/ml (630 μg/ml); and 5% susceptible at ≤0.72 mg/ml (720 μg/ml). Since all of the strains tested (99%) were susceptible at a concentration of ≤0.72 mg/ml, this suggests that EGC might be a good inhibitor of bacterial growth. **Table 2** shows the MIC data for total green tea extract and for the EGC component.

The data was collected by *in vitro* experiments, but the effect can be described using information that is known about the metabolism of EGC from green tea. The amount of green tea polyphenols that would be present in the urine, including the amount of EGC, will vary according to the origin of the tea. It has been found, for instance, that Japanese tea (an average of 15 teas) contains approximately 20 mg of EGC per gram of dry tea (Vuong et al., 2011). An average cup of Japanese green tea is made with one tablespoon of dry tea (instructions from package of tea) which is equivalent to approximately 7.5 g of dry tea (a package of 60 g of dry tea makes eight cups). That equates to approximately 150 mg of EGC per cup of Japanese green tea.

Urinary excretion after a single ingested dose of EGC has been shown to peak at 8 h after ingestion, with levels in the urine reaching to 3.0–5.0 mg (Yang et al., 1998). The amount of EGC excreted after ingestion of one cup of tea (as described above) should equal approximately 3.5 mg. Since the projected MICs for EGC are well below 3.5 mg, this suggests that even one cup of green tea could have an effect on a urinary tract pathogen, and drinking multiple cups over the course of a day could possibly provide a prolonged effect. Additional studies testing the *in vivo* effect of drinking green tea on UTIs could be useful for determining if the effects observed in this study have medical significance.

Studies have shown that concentrations of 500 μg of tea polyphenols can inhibit the growth of *E. coli*, and that concentrations of ≥5000 μg are considered bactericidal. This effect is believed to be due to the fact that tea polyphenols down regulate the production of proteins such as EF-2 (elongation factor for protein translation); proteins involved in phospholipid, carbon, and energy metabolism; and production of proteins involved in amino acid biosynthesis (Cho et al., 2007).

There was no correlation between MIC values in this study and the antimicrobial resistance of the isolates. Each level of MIC contained a variety of antimicrobial resistance patterns.

Another set of studies that would be interesting and potentially useful would be to test EGC and several of the standard antimicrobial agents used to treat UTIs to determine if there might be synergism between EGC and any of the antimicrobial agents. Studies have been done that show the ability of green tea to act synergistically with gentamicin and amikacin against *E. coli* (Neyestani et al., 2007).

## CONCLUSION

The results of this study have shown that green tea can have an antimicrobial effect on *E. coli* bacteria that cause UTIs. This is the first time that green tea has been reported to have this kind of effect. The data also adds to the current information on the potential health benefits of green tea. It is our hope that these findings will encourage further studies on the antimicrobial potential of green tea and other plant components.

## Conflict of Interest Statement

The authors declare that the research was conducted in the absence of any commercial or financial relationships that could be construed as a potential conflict of interest.

## References

[B1] Anderson G. G. Dodson K. W. Hooton T. M. Hultgren S. J. (2004a). Intracellular bacterial communities of uropathogenic *Escherichia coli *in urinary tract pathogenesis. *Trends Microbiol.* 12 424–430 10.1016/j.tim.2004.07.005 15337164

[B2] Anderson G. G. Martin S. M. Hultgren S. J. (2004b). Host subversion by formation of intracellular bacterial communities in the urinary tract. *Microbes Infect.* 6 1094–1101 10.1016/j.micinf.2004.05.023 15380779

[B3] Cabrera C. Artacho R Giménez R. (2006). Beneficial effects of green tea – a review. *J. Am. Coll. Nutr.* 25 79–99 16582024 10.1080/07315724.2006.10719518

[B4] Carson C. Naber K. G. (2004). Role of fluoroquinolones in the treatment of serious bacterial urinary tract infections. *Drugs* 64 1359–1373 10.2165/00003495-200464120-00007 15200349

[B5] Chacko S. M. Thambi P. T. Kuttan R. Nishigaki I. (2010). Beneficial effects of green tea: a literature review. *Chin. Med.* 5 13 10.1186/1749-8546-5-13 PMC2855614 20370896

[B6] Cho Y. S. Schiller N. L. Kahng H. Y. Oh K. H. (2007). Cellular responses and proteomic analysis of *Escherichia coli* exposed to green tea polyphenols. *Curr. Microbiol.* 55 501–506 10.1007/s00284-007-9021-8 17874165

[B7] Dias R. C. S. Marangoni D. V. Smith S. P. Alves E. M. Pellegrino F. L. P. C. Riley L. W. (2009). Clonal composition of *Escherichia coli* causing community-acquired urinary tract infections in the State of Rio de Janeiro, Brazil. *Microb. Drug Resist.* 15 303–308 10.1089/mdr.2009.0067 19857137 PMC3145954

[B8] Friedman M. (2007). Overview of antibacterial, antitoxin, antiviral, and antifungal activities of tea flavonoids and teas. *Mol. Nutr. Food Res.* 51 116–134 10.1002/mnfr.200600173 17195249 PMC7168386

[B9] George D. B. Manges A. R. (2010). A systematic review of outbreak and non-outbreak studies of extraintestinal pathogenic *Escherichia coli* causing community-acquired infections. *Epidemiol. Infect.* 138 1679–1690 10.1017/S0950268810001639 20642873

[B10] Gradišar H. Pristovšek P. Plaper A. Jerala R. (2007). Green tea catechins inhibit bacterial DNA gyrase by interaction with its ATP binding site. *J. Med. Chem.* 50 264–271 10.1021/jm0608170 17228868

[B11] Gündoğdu A. Long Y. B. Vollmerhausen T. L. Katouli M. (2011). Antimicrobial resistance and distribution of sul genes and integron-associated intl genes among uropathogenic *Escherichia coli* in Queensland, Australia. *J. Med. Microbiol.* 60(Pt 11) 1633–1642 10.1099/jmm.0.034140-0 21737542

[B12] Jadhav S. Hussain A. Devi S. Kumar A. Parveen S. Gandham N. (2011). Virulence characteristics and genetic affinities of multiple drug resistant uropathogenic *Escherichia coli* from a semi urban locality in India. *PLoS ONE* 6:e18063. 10.1371/journal.pone.0018063 PMC3064663 21464963

[B13] Kahlmeter G. (2003). Prevalence and antimicrobial susceptibility of pathogens in uncomplicated cystitis in Europe. The ECO.SENS study. *Int. J. Antimicrob. Agents* 22(Suppl. 2) 49–52 10.1016/S0924-8579(03)00229-2 14527771

[B14] Kaper J. B. Nataro J. P Mobley H. L. T. (2004). Pathogenic *Escherichia coli*. *Nat. Rev. Microbiol*. 2 123–140 10.1038/nrmicro818 15040260

[B15] Karlowsky J. A. Kelly L. J. Thornsberry C. Jones M. E. Sahm D. F. (2002). Trends in antimicrobial resistance among urinary tract infection isolates of *Escherichia coli* from female outpatients in the United States. *Antimicrob. Agents Chemother.* 46 2540–2545 10.1128/AAC.46.8.2540-2545.2002 12121930 PMC127340

[B16] Kau A. L. Hunstad D. A. Hultgren S. J. (2005). Interaction of uropathogenic *Escherichia coli* with host uroepithelium. *Curr. Opin. Microbiol.* 8 54–59 10.1016/j.mib.2004.12.001 15694857

[B17] Kucheria R. Dasgupta P. Sacks S. H. Khan M. S. Sheerin N. S. (2005). Urinary tract infections: new insights into a common problem. *Postgrad. Med. J.* 81 83–86 10.1136/pgmj.2004.023036 15701738 PMC1743204

[B18] Landgren M. Odén H. Kühn I. Osterland A. Kahlmeter G. (2005). Diversity among 2481 *Escherichia coli* from women with community-acquired lower urinary tract infections in 17 countries. *J. Antimicrob. Chemother.* 55 928–937 15886265 10.1093/jac/dki122

[B19] Lee M. J. Maliakal P. Chen L. Meng X. Bondoc F. Y. Prabhu S. (2002). Pharmacokinetics of tea catechins after ingestion of green tea and (-)-epigallocatechin-3-gallate by humans: formation of different metabolites and individual variability. *Cancer Epidemiol. Biomarkers Prev.* 11(Pt 1) 1025–1032 12376503

[B20] Li D. Liu B. Chen M. Guo D. Guo X. Liu F. (2010). A multiplex PCR method to detect 14 *Escherichia coli* serogroups associated with urinary tract infections. *J. Microbiol. Methods* 82 71–77 10.1016/j.mimet.2010.04.008 20434495

[B21] Luo H. Tang L. Tang M. Billam M. Huang T. Yu J. (2006). Phase IIa chemoprevention trial of green tea polyphenols in high-risk individuals of liver cancer: modulation of urinary excretion of green tea polyphenols and 8-hydroxydeoxyguanosine. *Carcinogenesis* 27 262–268 10.1093/carcin/bgi147 15930028

[B22] Mak J. C. (2012). Potential role of green tea catechins in various disease therapies: progress and promise. *Clin. Exp. Pharmacol. Physiol*. 39 265–273 10.1111/j.1440-1681.2012.05673.x 22229384

[B23] Marrs C. F. Zhang L. Foxman B. (2005). *Escherichia coli* mediated urinary tract infections: are there distinct uropathogenic *E. coli* (UPEC) pathotypes? *FEMS Microbiol. Lett.* 252 183–190 10.1016/j.femsle.2005.08.028 16165319

[B24] McKay D. L. Blumberg J. B. (2002). The role of tea in human health: an update. *J. Am. Coll. Nutr*. 21 1–13 11838881 10.1080/07315724.2002.10719187

[B25] Molina-López J. Aparicio-Ozores G. Ribas-Aparicio R. M. Gavilanes-Parra S. Chávez-Berrocal M. E. Hernández-Castro R. (2011). Drug resistance, serotypes, and phylogenetic groups among uropathogenic *Escherichia coli* including O25-ST131 in Mexico City. *J. Infect. Dev. Ctries.* 5 840–849 10.3855/jidc.1703 22169782

[B26] Muratani T. Matsumoto T. (2004). Bacterial resistance to antimicrobials in urinary isolates. *Int. J. Antimicrob. Agents* 24(Suppl.1) S28–S31 10.1016/j.ijantimicag.2004.02.001 15364302

[B27] National Institute of Diabetes and Digestive and Kidney Diseases. (2005). *Urinary Tract Infection in Adults*. Publication No. 06-2097. Bethesda, MD: National Kidney and Urologic Diseases Information Clearinghouse, National Institutes of Health

[B28] Neyestani T. R. Khalaji N. Gharavi A. (2007). Selective microbiologic effects of tea extract on certain antibiotics against *Escherichia coli* in vitro. *J. Altern. Complement. Med.* 13 1119–1124 10.1089/acm.2007.7033 18166124

[B29] Oliveira F. A. Paludo K. S. Arend L. N. V. S. Farah S. M. S. S. Pedrosa F. O. Souza E. M. (2011). Virulence characteristics and antimicrobial susceptibility of uropathogenic *Escherichia coli* strains. *Genet. Mol. Res.* 10 4114–4125 10.4238/2011.October.31.5 22057993

[B30] Pichon C. Héchard C. du Merle L. Chaudray C. Bonne I. Guadagnini S. (2009). Uropathogenic *Escherichia coli* AL511 requires flagellum to enter renal collecting duct cells. *Cell Microbiol*. 11 616–628 10.1111/j.1462-5822.2008.01278.x 19134121

[B31] Sahm D. F. Thornsberry C. Mayfield D. C. Jones M. E. Karlowsky J. A. (2001). Multidrug-resistant urinary tract isolates of *Escherichia coli*: prevalence and patient demographics in the United States in 2000. *Antimicrob. Agents Chemother.* 45 1402–1406 10.1128/AAC.45.5.1402-1406.2001 11302802 PMC90480

[B32] Schaeffer A. J. Klumpp D. J. Weiser A. C. Sengupta S. Forrestal S. G. Batler R. A. (2004). Infectious response to *E. coli*: molecular and genetic pathways. *Int. J. Antimicrob. Agents* 24S S57–S60 10.1016/j.ijantimicag.2004.02.011 15364309

[B33] Song J. M. Seong B. L. (2007). Tea catechins as a potential alternative anti-infectious agent. *Expert Rev. Anti. Infect. Ther*. 5 497–506 10.1586/14787210.5.3.497 17547513

[B34] Stenutz R. Weintraub A. Widmalm G. (2006). The structures of *Escherichia coli* *O*-polysaccharide antigens. *FEMS Microbiol. Rev.* 30 382–403 10.1111/j.1574-6976.2006.00016.x 16594963

[B35] Taguri T. Tanaka T. Kouno I. (2004). Antimicrobial activity of 10 different plant polyphenols against bacteria causing food-borne disease. *Biol. Pharm. Bull*. 27 1965–1969 10.1248/bpb.27.1965 15577214

[B36] Taguri T. Tanaka T. Kouno I. (2006). Antibacterial spectrum of plant polyphenols and extracts depending upon hydroxyphenyl structure. *Biol. Pharm. Bull*. 29 2226–2235 10.1248/bpb.29.2226 17077519

[B37] Taylor P. W. Hamilton-Miller J. M. T. Stapleton P. D. (2005). Antimicrobial properties of green tea catechins. *Food Sci. Technol. Bull*. 2 71–81 19844590 10.1616/1476-2137.14184 PMC2763290

[B38] Vosti K. L. (2007). A prospective, longitudinal study of the behavior of serologically classified isolates of *Escherichia coli* in women with recurrent urinary tract infections. *J. Infect*. 55 8–18 10.1016/j.jinf.2007.01.006 17331583

[B39] Vuong Q. V. Nguyen V. Golding J. B. Roach P. D. (2011). The content of bioactive constituents as a quality index for Vietnamese teas. *Int. Food Res. J.* 18 329–336

[B40] Wagenlehner F. M. Naber K. G. (2005). Fluoroquinolone antimicrobial agents in the treatment of prostatitis and recurrent urinary tract infections in men. *Curr. Infect. Dis. Rep.* 7 9–16 10.1007/s11908-005-0018-9 15610666

[B41] Wang Y. Ho C. T. (2009). Polyphenolic chemistry of tea and coffee: a century of progress. *J. Agric. Food. Chem*. 57 8109–8114 10.1021/jf804025c 19719133

[B42] Warren J. W. Abrutyn E. Hebel J. R. Johnson J. R. Schaeffer A. J. Stamm W. E. (1999). Guidelines for antimicrobial treatment of uncomplicated acute bacterial cystitis and acute pyelonephritis in women. Infectious Diseases Society of America (IDSA). *Clin. Infect. Dis*. 29 745–758 10.1086/520427 10589881

[B43] Yam T. S. Shah S Hamilton-Miller J. M. T. (1997). Microbial activity of whole and fractionated crude extracts of tea (*Camellia sinensis*), and of tea components. *FEMS Microbiol. Lett.* 152 169–174 10.1111/j.1574-6968.1997.tb10424.x 9228784

[B44] Yang C. S. Chen L. Lee M. J. Balentine D. Kuo M. C. Schantz S. P. (1998). Blood and urine levels of tea catechins after ingestion of different amounts of green tea by human volunteers. *Cancer Epidemiol. Biomarkers Prev*. 7 351–354 9568793

[B45] Zhanel G. G. Hisanaga T. L. Laing N. M. DeCorby M. R. Nichol K. A. Weshnoweski B. (2006). Antibiotic resistance in *Escherichia coli* outpatient urinary isolates: final results from the North American Urinary Tract Infection Collaborative Alliance (NAUTICA). *Int. J. Antimicrob. Agents* 27 468–475 10.1016/j.ijantimicag.2006.02.009 16713191

